# Chitosan-Based Composites: Development and Perspective in Food Preservation and Biomedical Applications

**DOI:** 10.3390/polym15153150

**Published:** 2023-07-25

**Authors:** Akash Kumar, Sangeeta Yadav, Jhilam Pramanik, Bhagavathi Sundaram Sivamaruthi, Titilope John Jayeoye, Bhupendra G. Prajapati, Chaiyavat Chaiyasut

**Affiliations:** 1 Department of Food Technology, SRM University, Sonipat 131029, India; 2 MM Institute of Hotel Management, Maharishi Markandeshwar (Deemed to be University), Mullana 133207, India; 3 Department of Food Technology, Guru Jambheshwar University of Science and Technology, Hisar 125001, India; 4 Department of Food Technology, William Carey University, Shillong 793019, India; 5 Office of Research Administration, Chiang Mai University, Chiang Mai 50200, Thailand; sivamaruthi.b@cmu.ac.th; 6 Innovation Center for Holistic Health, Nutraceuticals, and Cosmeceuticals, Faculty of Pharmacy, Chiang Mai University, Chiang Mai 50200, Thailand; 7 Department of Chemistry, Faculty of Science, Chulalongkorn University, Bangkok 10330, Thailand; 8 Shree S. K. Patel College of Pharmaceutical Education and Research, Ganpat University, Mehsana 384012, India

**Keywords:** fabrication, chitosan, nanocomposites, antimicrobial, polysaccharide

## Abstract

Chitin, which may be the second-most common polymer after cellulose, is the raw material of chitosan. Chitosan has been infused with various plant extracts and subsidiary polymers to improve its biological and physiological properties. Chitosan’s physicochemical properties are enhanced by blending, making them potential candidates that can be utilized in multifunctional areas, including food processing, nutraceuticals, food quality monitoring, food packaging, and storage. Chitosan-based biomaterials are biocompatible, biodegradable, low toxic, mucoadhesive, and regulate chemical release. Therefore, they are used in the biomedical field. The present manuscript highlights the application of chitosan-based composites in the food and biomedical industries.

## 1. Introduction

**Figure 1:**
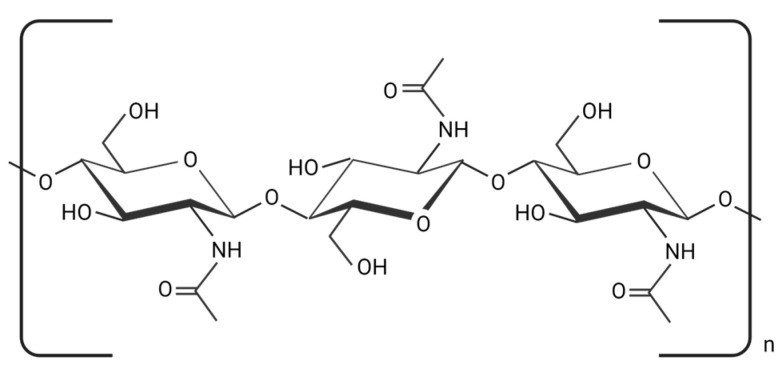
The chemical structure of chitin (created using BioRender.com on 4 July 2023).

Chitin is made by joining N-acetyl glucosamine residues through β (1–4) glycosidic linkages and is the second-most prevalent natural polysaccharide after cellulose [1,2]. Chitin (Figure 1) is a naturally occurring structured crystalline microfibril in arthropods’ exoskeletons, fungi, and yeast cell walls. The main commercial sources of α-chitin are crabs and prawn shells [3]. Squid comprises the β-form chitin, which is highly sensitive to deacetylation and has stronger reactivity and solubility, as well as more affinity to solvents and better swelling capacity than α-chitin [4]. The γ-form of chitin is generally found in fungi and yeast [5].

**Figure 2:**
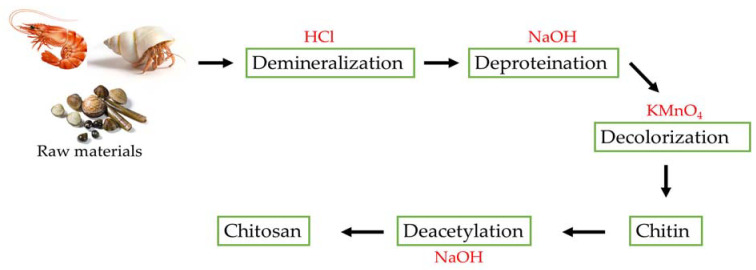
The steps in the synthesis of chitosan.

N-acetylglucosamine and glucosamine residues are copolymerized to form chitosan. It can be produced by deacetylating chitin using strong alkalis, which hydrolyzes the acetamide groups. Several chemical processes are involved in converting chitin into chitosan (CH). Demineralization, deproteinization, and deacetylation are the main steps in extracting chitosan (Figure 2).

Chitosan and its derivatives exhibit anti-hypoxic, adaptogenic, immunostimulant, antiviral, antibacterial, radioprotective, and hemostatic properties. In addition, they are nontoxic, biocompatible, and biodegradable [6,7]. Since chitosan sulfate possesses anticoagulant properties, it could be used to produce drugs that have an anticoagulant effect [8,9]. Further, sulfated chitosan acts as an antioxidant [10]. Chitosan enhances the amount of insulin and is a potential way to manage diabetes [11,12]. Chitosan in immunotherapy is recommended as a potential anticancer drug that inhibits the development of infections and tumor cells while promoting humoral and cellular immunity [13,14]. Chitosan-based materials are used to treat wounds and promote the growth of granulation tissue and fibroblasts [15].

**Figure 3:**
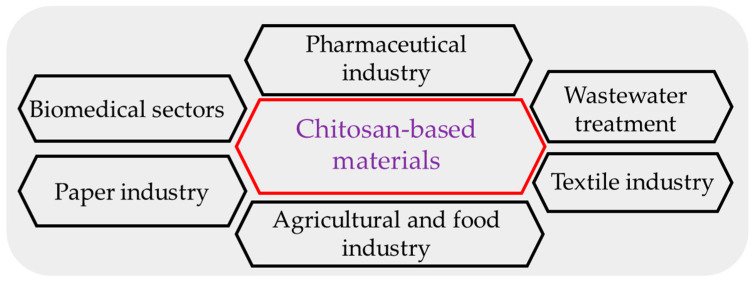
The application of chitosan in different sectors.

Chitosan products may create transparent films to enhance foods’ quality and shelf life. They are extremely dense and have antibacterial properties due to active amino groups [16,17]. Chitosan may be utilized to make edible films that minimize water loss and delay ripening due to its ability to build a durable, flexible, and partially permeable film. These qualities make chitosan unique from other edible coatings [18,19]. Apart from the above-stated advantages, chitosan-based films have disadvantages, including poor UV-light barrier features and mechanical attributes. Chitosan films’ hydrophilicity renders them incredibly vulnerable to moisture. Therefore, different kinds of natural substances, such as phenolic compounds, essential oils, plant extracts, or other biopolymers, can be added to improve the mechanical, physical, and biological properties of chitosan-based films [20]. CH-based materials are employed in various industries (Figure 3), including food, medicine, agriculture, and cosmetics [21,22]. This article emphasizes various approaches for fabricating chitosan-based composite food packaging systems and discusses the applications of chitosan-based materials in food preservation and biomedical science.

## 2. Fabrication Methods for Composite Films/Coatings

### 2.1. Solution Casting

**Figure 4:**
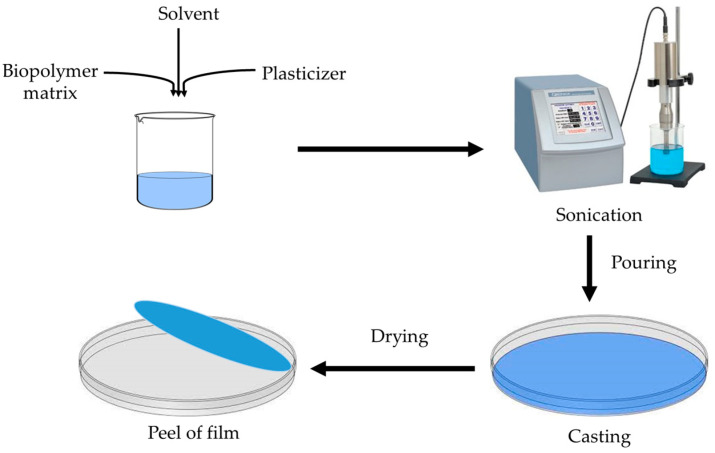
The solution-casting method of film formation.

This process is a commonly utilized method to produce chitosan coatings and films. First, it is dissolved in a solution, then the mixture is evaporated [23]. The process is affordable and easy, and the polymer structure is produced due to intermolecular electrostatic and hydrogen bonding [24]. However, the film becomes brittle due to this intermolecular entanglement. To enhance the mechanical characteristics of the films, several plasticizers (such as sugars, sorbitol, and glycerol) are added [25]. It is quite challenging to use this method on a commercial scale, and for this, there is still a need for further improvement. There are various steps in the fabrication process: Chitosan is first dissolved in an acid solution (pH less than 6.0), then it is blended, mixed, or crosslinked with other biopolymers, fillers, and functional materials at a different proportion, then the mixture is stirred to obtain a homogeneous viscous solution, then it is filtered, sonicated, or centrifuged to remove any air bubbles and insoluble particles. After this, the solution is cast or poured onto the surface for drying. After complete drying, the film is peeled off. Films are often created using the solution-casting process on a laboratory scale; however, further study is required to determine if it would be feasible to utilize solution-casting at a large scale [26,27]. The general method to fabricate the film from the solution casting method is shown in Figure 4.

The cast films of the chitosan blend and fucose-rich exopolysaccharide showed excellent characteristics such as biodegradability, antibacterial activity, and gas barrier [28]. As a result, the film may be used as a packaging material that extends the shelf life of foods.

### 2.2. Layer-by-Layer Assembly

**Figure 5:**
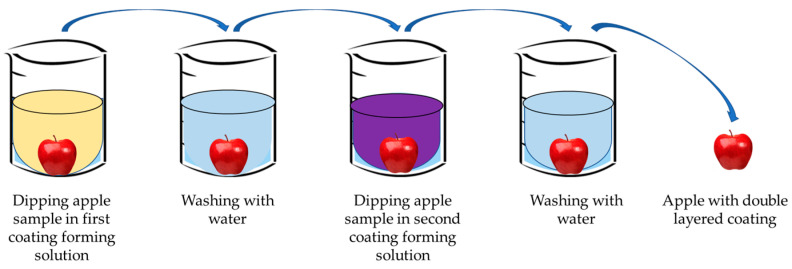
Layer-by-layer method of coating foods.

Due to its versatility and ability to integrate the functional features of various polymers, layer-by-layer assembly has been widely studied in producing nanocomposite films for effectively managing material properties and functionality. It is a technique for making multi-layered films that do not require any complicated equipment. In layer-by-layer assembly, surface modification primarily depends on the mutual attraction and deposition of alternating polyelectrolytes [29,30]. Artificial polymers, polysaccharides, proteins, and other biopolymers that carry net charge can be considered polyelectrolytes. Deposition can be accomplished by spraying solutions onto the substrate or submerging them in different polyelectrolyte solutions. Both approaches have the potential for scalability. Layer-by-layer deposition may create active packaging films and coatings by incorporating the active agents [31,32]. The general method to fabricate the coating on food materials through the layer-by-layer method is shown in Figure 5. It has been claimed that the layer-by-layer method, combined with other techniques, successfully preserves food quality and extends shelf life [33].

### 2.3. Extrusion

**Figure 6:**
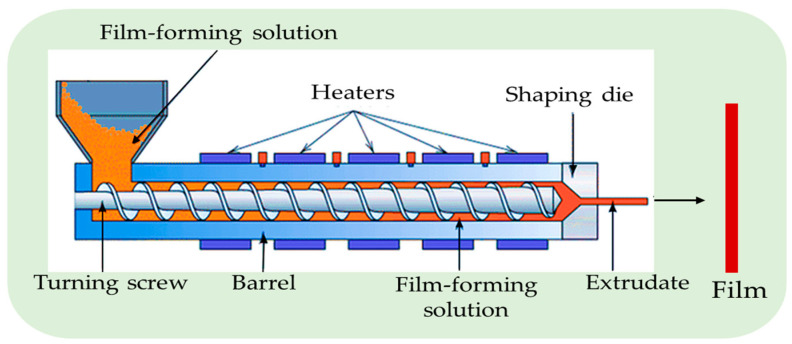
The extrusion method for the preparation of the film (adapted and recreated from Alsadi et al. [36]).

Most commercial plastic packaging films are made via extrusion techniques. Due to fast fabrication time and less energy-intensive nature, extrusion is frequently chosen over solution casting techniques [34,35]. The extrusion process includes several steps, such as preparing and mixing raw materials. Blending the mixture in an extruder for palettization, cutting the extrudates into pellets via a pelletizer, then drying the pellets and extruding them into the sheets via another extruder. Finally, film extruders blow the mixed resins into a film (Figure 6).

The films manufactured through extrusion have adequate mechanical and thermal characteristics. It is a potential method for film manufacture, but more research must be conducted to form chitosan-based films via this technique [37]. The extrusion method produces an antimicrobial film from a mixture of chitosan, starch, and poly (lactic acid) to preserve food with high water activity [38].

### 2.4. Coatings (Spraying, Dipping, or Spreading)

**Figure 7:**
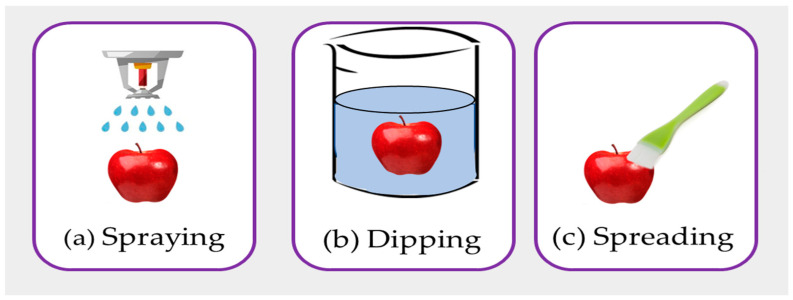
The different methods of coating such as (**a**) spraying, (**b**) dipping, and (**c**) spreading.

The coating can be applied to the skin to extend the shelf life of fresh items like fruits, vegetables, fish, meat, etc. [39]. The most common methods of food coating are spraying, dipping, or spreading (Figure 7). With a few instruments, such as a brush or spatula, spread coating is accomplished. The coating is a useful method to prevent microbial growth, enhance the shelf life, and maintain quality keeping [40].

The coating procedure entails several steps:

1. Preparation of raw materials through mixing the right proportions of chitosan and fillers.
2. Preparation of coating samples through various methods such as irradiating, heating, mixing, and steam flash pasteurizing.
3. Sanitizing of the food samples through sodium hypochlorite.
4. Application of the chitosan-based composite solutions to food through the sterile spreader.
5. Drying under specific circumstances.
6. Packaging and storage in suitable conditions [41].

Antimicrobial components may migrate from the films to the food to increase the shelf life of food [42]. In an active packaging system, the coating is formed by dipping or spraying methods. In dipping methods, food is dipped into a previously made acidic chitosan solution that also contains preservatives and plasticizers to enhance the efficiency of the coating or film [43]. The coating process typically involves dipping the food for a short period in the composite solutions, draining the excess solution, and then drying the samples. Food products have been coated with one or more layers using various dipping techniques. However, double-dipping was preferred over three or more dipping in certain circumstances. This method is easy and affordable because there is no need for complicated equipment, and it gives high preservation efficiency. Similar procedures are followed for spray coating, except spraying is conducted with a sprayer supported by compressed air [44]. While dipping and spraying processes are easy, economical, and often used in most food manufacturing lines, these methods can hinder the food’s sensory qualities [45]. Therefore, it is strongly advised to employ this approach carefully to apply chitosan-based biopolymer coatings or films. These methods are used for coating fruits and vegetables to improve their shelf life [46].

### 2.5. Crosslinking

**Figure 8:**
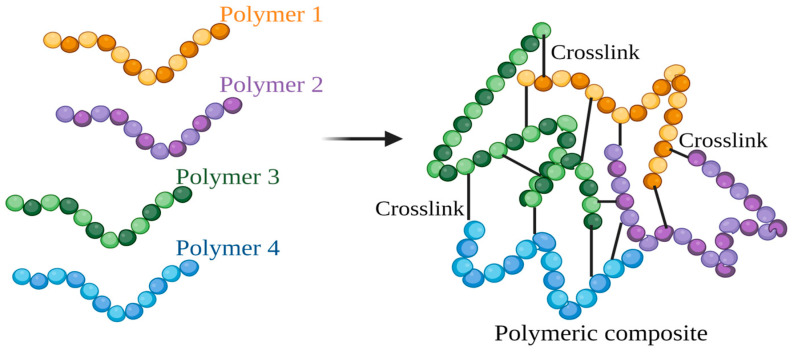
The schematic of the crosslinking for developing polymeric composite (created using BioRender.com on 4 July 2023).

Two polymers are linked together via a crosslink developed by a chemical process, covalent or ionic bonds, or weaker bonding interactions [47] (Figure 8). The component polymers in the crosslinked composite may exhibit new characteristics when combined while retaining their unique characteristics. An interpenetrating polymer network (IPN) is one sort of crosslink that has been extensively studied. Among other chemical substances, cross-linkers include polymers [48], oxides [49], metals [50], and amino acids [51]. Various polymers may successfully create an IPN with the chitin-based material, including polymers made of epoxides, alcohols, and carboxylic acids. However, some of these polymers may affect membrane development, making their usage undesirable [52]. Crosslinking techniques can be used to manufacture food packaging films. The food packaging film was developed using a mixture of chitosan and gelatin with genipin as a crosslinked filler and a hybridization of quercetin and rosemary essential oil. The film has excellent UV protective, antioxidant, and antibacterial properties. Thus, the developed film can be employed as active food packaging materials [53].

### 2.6. Electrospinning

**Figure 9:**
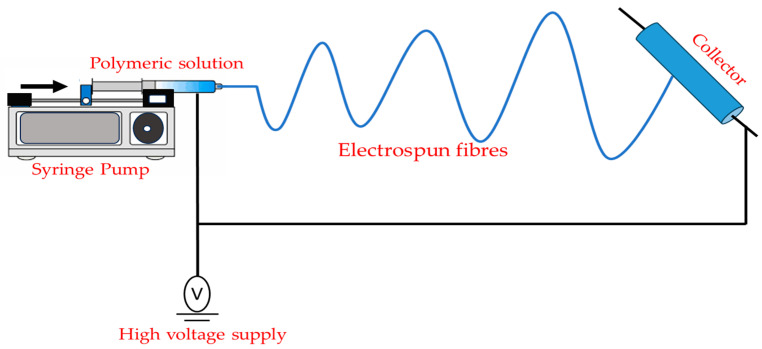
Schematic representation of the fundamental electrospinning arrangement.

A spinneret, a collector (a grounded wire), and a high-voltage power source comprise a simple electrospinning system (Figure 9). Spinneret (which contains polymer solution) has a blunt-tip needle and a syringe pump that regulates the flow of the polymer solution [54]. To control the structure of electrospun nanofibers (coaxial setup for core-sheath and hollow nanofibers [55]) or to increase the throughput of electrospun nanofibers (multi-needle electrospinning setup [56]), numerous advanced electrospinning setups have been developed [57]. Typically, the electrospinning procedure may be broken down into four steps:

(1) Formation of the cone-shaped jet from a pendant droplet by charging it in an electric field.
(2) Lengthening of the charged jet.
(3) Stretching and thinning of the charged jet under the electric field causes bending instability.
(4) Solidification and collection of the jet as solid fibers on a grounded collector [54].

The development of chitosan-based fibers for wound healing will largely depend on incorporating suitable copolymers. However, electrospinning still has technical difficulties producing chitosan-based fibers [58].

This section summarizes the various fabrication methods used (such as solution-casting, layer-by-layer, extrusion, coating, crosslinking, and electrospinning) to create chitosan-based materials that may be used in the biomedical and food industries. Fabrication methods can be used to develop a chitosan-based composite to enhance the mechanical, UV-shielding, antibacterial, and antioxidant properties. As a result, chitosan composites are suggested as potential materials to be utilized in the biomedical and food sectors.

## 3. Improvisation of Chitosan-Based Composites

Films and coatings made from chitosan have a few drawbacks, such as poor water resistance, reduced mechanical characteristics, and poor ultraviolet light barrier properties [20,59]. To overcome these problems, chitosan may be mixed with organic biopolymers, including polysaccharides and proteins. The blend of chitosan and polysaccharide blends often offers several benefits over other biopolymer blends, including the inexpensive, stable, and enhanced characteristics of the generated films [47]. Chitosan may also blend with polyphenols, plant extracts, polyvinyl alcohol, metal/metal oxide nanoparticles, and clay [60].

Chitosan-based composite films have been studied for their physical, mechanical, and biological characteristics. For example, a compact structure polymer composite was made when corn starch was blended with chitosan. The composite films had improved elongation, tensile strength, and other mechanical properties. They reduced water vapor permeability, an important film characteristic for packaging used in the food sector [61].

The composite film of starch and chitosan blend has improved biological, mechanical, and physical characteristics. The chitosan-starch films showed antibacterial action against *Listeria innocua*, indicating that these composites might be utilized in the packaging sector to guarantee the safety of food items [62]. Kaya et al. described a blend of sporopollenin-chitosan and the study suggested that the higher amounts of sporopollenin in chitosan-based films were beneficial in terms of improved physical, chemical, and mechanical characteristics, hydrophobicity, and biological properties [63].

Through the blending of pectin and chitosan, a biodegradable packaging material was made that could be used for food packing. The composite film has a lower water vapor transmission rate than the sole pectic film. Similarly, composite film has improved tensile strength and transparency [64].

Phenolic chemicals exhibit synergistic effects and improve the antibacterial and antioxidant activities of composite films when blended with chitosan [59]. The existence of the crystalline phase, the type of chitosan matrix, the microstructure of the chitosan network, and intramolecular bonding all have a significant role in the mechanical properties of the film [65,66]. According to the literature, different polyphenols have different effects on the mechanical properties of films; for example, the tensile strength (TS) and elongation at the break (EAB) of the chitosan-*propolis* composite film are increased by the addition of *propolis* extract [67]. The blending of gallic acid [68], epigallocatechin gallate nanocapsules [69], ellagic acid [70], protocatechuic acid [71], proanthocyanidins [72], syringic acid [73], phenolic acids [21], or curcumin [74] into chitosan-based films increases the resulting composite’s mechanical strength.

In chitosan-based composite films, blending of olive leaf extract [75] or purple rice and black rice extracts [76] provides an improvement in TS and EAB; however, adding *Punica granatum* peel extract [77], *Thymus vulgaris* extract [78], *Curcuma longa* extract [79], *Mangifera indica* leaf extract [80], or purple-fleshed sweet potato extract [81] only improved the TS; additionally, adding *Punica granatum L.* extract [82], *Citrus paradisi* seed extract [83], *Berberis crataegina* fruit extract [84], and *Nigella sativa* seedcake extract [85] improved only the elongation. In contrast, incorporating certain polyphenols may weaken the mechanical strength of the composite film. For example, blending Chinese chive root extract in chitosan reduces the tensile strength of the composite film [65]. Similarly, composite film incorporated with apple peel polyphenols exhibited lower tensile strength than controlled film [66]. Therefore, the blending of appropriate polyphenols plays an important role in the mechanical characteristics of the composite film.

Blending propolis in the chitosan matrix reduced the water vapor and oxygen transmission rates of the composite film [86]. The water vapor transmission rate was reduced when polyphenol-rich natural extracts (*Allium tuberosum* root extract [64], pomegranate peel extract [82], *Nigella sativa* seedcake extract [85], *Lycium barbarum* fruit extract [87], *Herba Lophatheri* extract [88], and olive leaves extract [75]) were blended with chitosan. The antioxidant efficacy of chitosan and phenolic compounds-based composite films is greater than ordinary ones [21,71].

Ammonium chitosan/polyvinyl alcohol composite film has reduced water vapor permeability with strong tensile strength [89]. The composite film of polyvinyl alcohol, chitosan, and different D-Limonene (DL) concentrations exhibited good tensile strength and low water vapor permeability. Additionally, the composite films exhibited antibacterial activity against *Staphylococcus aureus* and *Escherichia coli* [90]. Yu et al. found that adding silica to the polyvinyl alcohol/chitosan films might increase tensile strength by up to 45% by creating hydrogen bonds between silica and polyvinyl alcohol or chitosan. The addition of silica reduced the oxygen and moisture transmission rate and increased the shelf life by three times [91]. A composite film of silver nanoparticles/gelatin/chitosan had antibacterial capabilities against *Staph. aureus* and *E. coli,* as well as improved mechanical and water barrier characteristics [92]. The incorporation of ginger essential oil in nanocomposite films of chitosan and montmorillonite exhibited improved the film’s ability to block light, gases, and water vapor [93]. In addition, incorporating graphene oxide into the chitosan film improved the tensile strength and thermal properties [94]. The above-stated properties of chitosan composite proved that these composites may be utilized in the food industry.

## 4. Application of Chitosan-Based Composite in the Food Industry

### 4.1. Utilization of Chitosan-Based Composite in the Preservation of Seafood

Chitosan has been shown to prolong the shelf life of fish and fishery products by preventing the growth of spoiling bacteria and maintaining product quality [95]. During the 20 days of storage of grass carp fillets, chitosan coating prevented microbial growth and lipid degradation, decreased water loss, and maintained pH [96]. Additionally, it was found that the total viable and psychrotrophic counts were inhibited, resulting in less amine synthesis. Chitosan coating may reduce lipid oxidation by producing chelating-free ions [97]. Active substances can be incorporated into chitosan-based films to improve their ability for preservation [98]. A coating made of chitosan and curcumin nanoparticles dramatically reduced the bacteria count and maintained it within the allowable range in *Schizothoraxpranati* fillets. Chitosan and the curcumin-based film might squelch free radicals and protein degradation in fillets and successfully delay lipid oxidation and the production of volatile amines [99]. Red sea bream fillets were protected from oxidation, amine development, and total viable count by a chitosan nanoparticle-based coating containing cinnamon-perilla essential oil and anthocyanidins [100]. Red sea bream maintained at 4 °C benefited from active packaging made of chitosan that included ginger essential oil to prevent the growth of bacteria and lipid oxidation [101]. For 28 days of storage of shrimps, the chances of lipid oxidation were decreased by a film made of chitosan and tea polyphenols [102]. Additionally, the coatings made of chitosan postponed the shrimps’ proteolysis, an enzymatic process.

The shelf life of vacuum-packed rainbow trout was examined by Rezaeifar et al. [103] concerning the impact of chitosan coatings with lemon verbena extract and essential oil. The investigation showed that the treated samples had reduced the quantity of peroxide, total volatile basic nitrogen, and H_2_S-producing bacteria. The application of essential oil and extract of lemon verbena improved the fish’s sensory quality. Ehsani et al. studied the impact of chitosan films combined with sage essential oil on the deterioration of fish burgers made up of common carp flesh. According to the findings, coatings successfully inhibit or retard the growth of harmful and spoilage-causing bacteria [104]. The sage essential oil introduction prevents the production of off-flavor. Demircan et al. treated mackerel fillet with chitosan comprising ethyl lauroyl arginate and lemon essential oil. The results revealed that the samples treated with composite had more antioxidant capacity than the untreated samples [105]. According to Zamani et al., chitosan and cumin extract coating showed antibacterial and antioxidant characteristics and preserved the quality of rainbow trout fillets [106].

### 4.2. Utilization of Chitosan-Based Composite in the Preservation of Meat and Meat Products

Meats are perishable and more susceptible to pollutants that can enter the interior layers of muscle [107]. Once microbes have penetrated the inner layers, it is difficult to kill them by cooking [108]. Food oxidation and microbial spoilage are two significant issues that impact meat’s quality and shelf life. Packing materials are increasingly made with antimicrobials and antioxidants instead of artificial food preservatives.

Numerous studies have been conducted to find natural active compounds that can replace artificial antioxidants and antimicrobials because these are associated with potential health risks. Therefore, numerous studies have been conducted on producing chitosan-based films and utilizing them in processing meat products, including red meat, poultry, and pork [109]. Green tea extract has been included in a chitosan-based film as an active material to extend the shelf life of pork sausages [110]. The findings revealed that pork sausages wrapped in chitosan film containing green tea extract exhibited fewer changes in color, texture, 2-thiobarbituric acid value, sensory qualities, and microbial growth than the control. Finally, they proposed that adding green tea extract to chitosan film might improve its antioxidant and antibacterial qualities, preserve the quality, and extend the shelf life of pork sausages. In another research, a chitosan-based film was synthesized that included acetic acid. The impact of this film on the shelf life of meat was investigated. Results revealed that chitosan-acetic acid film could retain the freshness and quality of chilled meat for longer periods than low-density polyethylene (LDPE) film. The chitosan films successfully suppressed the microbial growth in chilled meat during storage. The findings showed that the antibacterial properties of the chitosan films on meat were mostly responsible for controlling microbial growth suppression [111].

Coating made from chitosan, pomegranate peel extract, and thymus essential oil increased the shelf life of fresh beef [112]. The findings of this study demonstrated that the coating successfully reduced bacterial numbers and the chance of lipid oxidation. Zhang et al. developed a coating made of chitosan, gelatin, and tarragon essential oil to increase the shelf life of pork slices stored in the refrigerator. The outcomes demonstrated that the coatings maintain the quality of the pork slices. When the skinned turkey breast fillet was coated with chitosan coatings consisting of oregano essential oil and grape seed extract, there was a fall in *Enterobacteriaceae* counts. The study also revealed that these coatings might lessen lipid oxidation [113]. Gaba et al. studied the effectiveness of chitosan films combined with oregano and thyme essential oils towards meat deterioration and harmful microorganisms. The manufactured films inhibited the psychrophilic bacteria’s ability to proliferate [114].

### 4.3. Utilization of Chitosan-Based Composite in the Preservation of Postharvest Foods

Most horticultural commodities have a relatively limited postharvest life, which means fresh agricultural produce is perishable. Even after harvest, fruits and vegetables continue to breathe and perspire, reducing their shelf life and degrading their freshness. Fruits change physiologically and biochemically during ripening, including the degradation of chlorophyll, the breakdown of cell walls by enzymes, changes in sugar content, changes in respiratory activity, the formation of ethylene, and variations in the amounts of aromatic compounds [115]. Reactive oxygen species can injure cells and degrade the quality of fruits and vegetables when released in excess or generated during electron transport in the mitochondria [116]. Many scientists have researched organic, nontoxic compounds that may be utilized to prolong the shelf-life of fruits and vegetables after harvest. In one of these studies, strawberry fruit was coated with an edible chitosan-based coating containing *Thymus capitatus* essential oil, which increased the fruit’s shelf life in cold storage by up to 15 days [117].

Biopolymer-based edible film wrapping or coating is cheap, easy, and efficient in preventing moisture loss and lowering the degradation and respiration pace. It has been discovered that chitosan-based composites combined with nanomaterials and natural antimicrobials are beneficial for extending shelf life and preserving the quality of postharvest products [118]. The inclusion of nano-fillers improves the physicochemical and biological characteristics of natural biopolymers. Fresh fruits and vegetables with chitosan-based coating showed less weight loss, firmer texture, more antioxidant activity, and soluble solids than the control [119]. Quaternized chitosan films with carboxymethyl cellulose increase the shelf life of whole bananas [120].

A study finds the impact of chitosan composite film with apple peel polyphenol on the shelf life of strawberries [121]. Compared to uncoated fruits, the coated fruits’ antioxidant levels remained merely steady. As a result, decreased antioxidant capacity during storage deteriorates the quality of fresh produce. Effective oxygen radical scavengers are directly connected to the capacity of chitosan-based apple peel polyphenol composite covering to prevent decay, decrease enzymatic activity, and maintain the high-quality characteristics of fruits, leading to the degradation of antioxidant chemicals [122]. Yage et al. method of inducing film formation on mangoes’ surfaces by covering them with chitosan/nano-titanium dioxide decreased deterioration and water loss while delaying respiratory peaks [123]. Red grapes’ freshness was investigated using a biodegradable, antibacterial chitosan starch composite film [124]. According to the findings, the biodegradable film might be used to preserve grapes because of its superior water retention and antibacterial effectiveness. In similar studies, Yang et al. utilized chitosan coating consisting of blueberry leaf extract to preserve the postharvest quality of fresh blueberries [125]. The composite film made from chitosan and plant extracts was rich in antioxidants [126]. The creation of a composite film made of chitosan and banana peel extract was studied. Different amounts of banana peel extract were incorporated into chitosan membranes. The study’s findings revealed that the chitosan-banana peel extract composite membranes exhibited high antioxidant activity [127]. Essential oils from plants, used to make antimicrobial composites for food packing and preservation, offer strong antioxidant activity and antibacterial capabilities [128]. According to a study, adding essential oils (clove, oregano, cinnamon, or eucalyptus oil) to chitosan increased antibacterial efficacy in food packages. Chitosan-based active packaging ideas were created because of its antibacterial action to limit, block, or postpone the growth of microbes and extend the product’s postharvest/post-manufacturing shelf life [129].

### 4.4. Utilization of Chitosan-Based Composite in the Monitoring of Freshness/Spoilage

Food quality is a crucial factor in the food supply chain. Several analytical techniques for evaluating food quality, including chromatography and mass spectrometry, are costly, complex, heavy, and immobile. A quick detection technique uses biosensors with great sensitivity, quick reaction times, compact sizes, low costs, and mobility. Furthermore, biosensors are frequently employed to identify nutritional components and dangerous ingredients in food [130]. Hydrogels constructed from chitosan are materials for the manufacture of biosensors and indicators. As a result, they have a broad spectrum of potential uses in the food manufacturing industry, such as evaluating food quality. pH value is a crucial indicator frequently used to assess the degree of food decomposition and freshness in the supply chain.

Based on pH, an effective, sensitive, and low-cost chitosan/corn starch hydrogel film-embedded extract from *Brassica oleraceae* was prepared as a pH visual indicator. It exhibits a distinct color as fish spoils due to its strong sensitivity to pH changes. Buyers can use a noticeable color shift to quickly indicate the fish’s quality in the packaging [131]. According to Ebrahimi et al., a hydrogel pH indicator of chitosan was made that contains anthocyanins and can be utilized to assess how fresh milk is. Lactic acid is formed during storage by the lactic acid bacteria, which causes the pH of the milk to fall. The findings demonstrated that the pH indicator, which serves as a reliable test of the freshness of milk, changed from blue to violet rose after 48 h of milk storage [132].

## 5. Application of Chitosan-Based Composite in the Biomedical Industry

Chitosan has been used in the biomedical industry for its various benefits, including minimal immunoreactions, strong biocompatibility, simple biological degradability, great mucoadhesion, and natural abundance [8]. However, chitosan’s limited water solubility is a problem that restricts its biomedical value, particularly under physiological circumstances where it is weakly soluble and has poor absorption [133]. Furthermore, chitosan has a very poor transfection efficiency and lacks numerous beneficial characteristics, significantly restricting its use range. To increase the water solubility of chitosan, it may be blended with other compounds that improve the potential of chitosan to be utilized in therapeutic applications [134].

Surprisingly, it has been shown that the blending of compounds with chitosan not only improves the structural characteristics but provides some new characteristics. Because of the distinctive structure of chitosan, it may go through various reactions including phosphorylation, crosslinking, reduction, oxidation, complexation, halogenation, and acylation, which are mainly responsible for new characteristics [135]. As a result, chitosan and its derivatives are well known for their adaptable biological and chemical properties. Compared to native chitosan, their functionalized analogs construct self-assembling nanostructures more readily and quickly than native chitosan. The construction of hydrophobic equivalents with amphiphilic qualities and chemical groups with various therapeutic and active substances increased the bioactivity and DNA complexing capabilities [136]. Chitosan was additionally identified as an antibacterial agent. It is hypothesized that chitosan increases the movement of Ca++ from anionic sites of the bacterial cell membranes, which causes cell damage. It also prevents plaque against *Porphyromonomas gingivalis*, *Prevotella intermedia*, and *Actinobacillus actinomycetemcomitans* [137,138]. While generally harmless to mammals, chitosan possesses a broad spectrum of action against gram-negative and gram-positive bacteria. The chitosan and its composite characteristics show their potential to be utilized in the biomedical sector, and a more thorough explanation is provided below.

### 5.1. Utilization of Chitosan-Based Products in Drug Delivery

Chitosan is a nontoxic and adsorbable polymer and reduces medication irritability; thus, it is preferred in drug administration [139]. Chitosan hydrogels have been extensively studied for their potential as a drug delivery method due to their biocompatibility, biodegradability, and capacity to regulate drug release. Chitosan hydrogel was used to encapsulate diclofenac sodium salt [140]. For topical use in wound healing, genipin crosslinked porous chitosan fiber has been produced [141]. To cure diabetes, chitosan hydrogels have been produced [142,143]. Different fatty acids, N-isopropyl acrylamide, and 2-acrylamide-2-methylpropane sulfonic acid are used to create a chitosan-amide-crosslinked matrix [142]. Phan et al. used a straightforward dropping method to form insulin-loaded hydrogel beads. The insulin was enclosed in stacked double hydroxides and covered with chitosan and alginate to make the hydrogel beads. The beads successfully protected insulin from acid, releasing it gradually in the small intestine [143]. Using the salt leaching method, metformin nanoparticles are blended into a chitosan/PVA polymeric composite [144]. This technique creates a porous structure that enables the medicine to be released under regulated conditions; the release rate is based on the size and distribution of the pores inside the composite. Chitosan has been used as bioadhesive beads to increase drug retention in the stomach while delivering medications to the stomach [145]. Chitosan hydrogels can be used as cancer therapy tools to deliver specific drugs. The hydrogels can be used to deliver anticancer medications such as cytarabine [146], methotrexate [147], 5-fluorouracil [148,149], doxorubicin [150,151], and cisplatin [152] directly to cancer cells. Li et al. created a thiolated chitosan hydrogel with a gelation point of 37 °C to target and treat a solid tumor. The negatively charged surface of the liposome electrostatically interacted with the positive charge of chitosan, extending the gelation period when the liposome-encapsulated curcumin was added [153].

### 5.2. Utilization of Chitosan-Based Products in Tissue Engineering

Chitosan composite-based scaffolds have been reported to be employed for tissue engineering during the past several years due to their cationic properties and ability to produce linked porous structures [154]. For bone repair and reconstruction, chitosan is coupled with other biomaterials like hydroxyapatite and bioactive glass-ceramic to generate a carbonated apatite layer that improves the structural integrity of the bone [155,156,157]. Chitosan-based composite can be utilized to enhance bone regeneration by delivering growth factors or medications directly to the location of the damage or by offering a biomimetic template for the formation of new bone tissue. Extracellular matrix (ECM) components are produced more readily when chitosan is present. ECMs like collagen are necessary for bone formation and repair [158]. Even though chitosan is a promising material for many medicinal purposes, it cannot be used to create bone tissue engineering. Chitosan lacks the osteoconductive qualities of real bones and is not robust enough to withstand the weight-bearing needs of bone implants. Therefore, chitosan has been combined with other biopolymers, including chitin, silk, and polycaprolactone, as well as bioactive nanoceramics like hydroxyapatite and zirconia, to generate bio-composites, which have been used by researchers to overcome these constraints [159,160]. These bio-composites are more suitable for bone tissue engineering applications because they have increased mechanical strength and structural integrity [161]. A dual network hydrogel for articular cartilage regeneration was created by Liu et al. using thiolated chitosan and silk fibroin. The composite had spontaneous gelling and excellent injectability in response to pH and temperature. Chitosan increased strength and stiffness, whereas silk fibroin was primarily responsible for elasticity. The platform supported chondrocyte proliferation and encouraged the deposition of chondrocyte-specific matrix [162].

Chitosan-based hybrid systems may be used for cardiac tissue engineering [163]. Human cardiac ECM, chitosan, and gelatin were used by Lv et al. to build a 3D scaffold for a tissue-engineered heart patch [164]. In a study, an injectable thermosensitive hydrogel made of chitosan, dextran, and β-glycerophosphate loaded with mesenchymal stem cells from the umbilical cord was described to treat myocardial infarction [165]. Mombini et al. created electrospun cardiac conductive scaffolds using polyvinyl alcohol, chitosan, and various concentrations of carbon nanotubes [166]. A polycaprolactone (PCL)/chitosan membrane was combined with chitosan nanoparticles (CSNPs) to provide a biodegradable scaffold for growing ocular cells. The scaffolds were brittle when they were dry but following a brief immersion in phosphate-buffered saline, they were flexible and simple to handle. Scaffolds with a CSNP/PCL ratio 50/25 exhibited the highest degree of transparency. The discovered scaffold was nontoxic and encouraged corneal epithelial cell proliferation [167].

### 5.3. Utilization of Chitosan-Based Products in Wound Healing

The appropriate wound dressing must be placed to protect the wound site from external mechanical and microbiological stress to speed up the healing process. Traditional dressings made of cotton, dressings, and gauze sometimes fall short of offering a moist and hospitable environment and might hurt when removed because of wound drainage [168]. Chitosan has the potential to be utilized in wound healing. The usage of chitosan-based biomaterials for wound healing applications has grown, either on their own or in conjunction with other organic or synthetic biomaterials. Chitosan-based biomaterials have been utilized for the dressing of wounds because of oxygen permeability and biocompatibility [169]. In wound healing, the impact of chitosan biomaterial on collagen production has been investigated [170]. In another study, silver nanoparticles have been functionalized with chitosan utilizing an ethanolic buds extract of *Sygyzium aromaticum*. The results revealed that at relatively high concentrations of CS-AgNP, there is a drop in fibrinogen level and a decrease in platelet aggregation. It has been demonstrated that CS-AgNP may be employed as an efficient antibacterial agent and anticoagulant with minimal toxicity in the biomedical sector [171]. A porous sponge-like dressings material was prepared by freeze-drying water-soluble adenine-modified chitosan derivative [172]. The intention was to address the problem of bacterial infections, which can obstruct healing and cause significant tissue damage during the early stages of wound healing. To create sticky chitosan hydrogels inspired by mussels, Sun et al. developed sticky chitosan hydrogels that exhibited outstanding mechanical strength and sustained dynamic adherence to biological surfaces and could be utilized as dressings material in the wound healing process. Chitosan-gallic acid hydrogel exhibited wound healing and hemostatic characteristic. Additionally, the hydrogel showed improved antibacterial activity against *E. coli* and *S. aureus* and high biocompatibility [173]. The application of various chitosan-based composites in the food and biomedical sector has been listed in Table 1.

## 6. Miscellaneous

Chitosan produces many nanostructures, such as nanoparticles, nanohydrogels, nanofibers, and nanocomposites. These structures are used effectively as nanocarriers for enclosing bioactive compounds [174]. It has been demonstrated that chitosan and its derivatives exhibit broad-spectrum antibacterial activity and can be used as an antimicrobial agent [175]. Chitosan can also be used as a food additive to improve product sensory quality and shelf life [176]. In addition to the abovementioned application, chitosan is a thickener, emulsifier, and substitute for low-calorie foods [177]. Chitosan tends to increase the viscosity of the continuous phase, making it more difficult for dispersed particles to diffuse and slowing the rate of droplet aggregation [178]. This quality is used while creating items like sauces, sweets, and ice cream. Chitosan may be used to clarify fruit juices [179,180], beer [181], and purify water [182]. Chitosan has been proposed as a functional additive for food and feed products [183]. Chitosan has several positive health effects, such as lowering blood cholesterol and blood pressure, scavenging reactive oxygen species, shielding against infections, controlling arthritis, enhancing anticancer properties, and regulating inflammation [183]. Chitosan can also be used as a carrier for encapsulating probiotic components because of its great biocompatibility, emulsifying capability, and hypoallergenic [178] and hypocholesterolemic [184] characteristics.

Gamma irradiation was used to create hydrogels based on various ratios of chitosan and sodium alginate, while glutaraldehyde served as a crosslinking agent. These hybrid hydrogels were discovered to have great water swelling and high heat stability. This research also examined the ketoprofen drug’s pH-sensitive release characteristics [185].

Chitosan is used as a drug delivery method for treating brain tumors and neurological illnesses. Due to various characteristics of chitosan nanoparticles, such as efficient absorption by tumor cells and nasal mucosa, regulated release, low toxicity, biodegradability, and biocompatibility, it has efficiently treated brain illnesses [186].

Chitosan composites may be used for treating burns, for the development of artificial kidneys, as a blood anticoagulating agent, and for tendon or blood vessel engineering [187]. Crosslinking processes modulate the three-dimensional structures of chitosan and increase chitosan utilization in the biomedical sector [187]. Separating and purifying physiologically active molecules using chitosan biopolymers because they include hydroxyl groups and electron-donating amino is possible. Recent research has shown that chitosan-based compounds are useful for preventing the growth of biofilms and reducing the virulence of certain harmful bacteria [188]. Chitosan acts as a dental adhesive, a component of dentifrices (toothpaste, chewing gum) with antibacterial properties, an agent to prevent dental disorders, an agent to increase salivary secretion, etc. [189], while adding suitable organic or inorganic elements might enhance the effects of chitosan on periodontal regeneration [190]. This is due to the complicated periodontal microenvironment, which necessitates a multi-component platform to accommodate its diverse compositional and mechanical requirements for effective regeneration [191]. Polymethyl methacrylate was modified with chitosan salts or chitosan-glutamate to create a novel antifungal denture base material [192]. To control the medication delivery systems, pH-sensitive polymeric materials were developed by sodium alginate, attapulgite, and chitosan-g-poly (acrylic acid) [193].

## 7. Conclusions

Chitin is the primary source of chitosan. Sources of chitin include the exoskeletons of crustaceans, green algae, fungus, and the cuticles of insects. Crustacean shells are the main source of chitin utilized in industry. Different chemical methods, including decalcification, deproteinization, decolorization, and deacetylation, are used in the commercial manufacturing of chitosan. Acidic and alkali treatments are damaging the environment. Therefore, there is a need to determine the biological methods that may be used to produce chitosan. Chitosan and its composites have the potential to be utilized in food preservation because of their various biological properties. However, its utilization is limited due to its poor solubility and poor mechanical characteristics. Chitosan composite films and coatings can improve the shelf life of foods by suppressing bacterial populations and deterioration reactions. Even though numerous studies on the development of chitosan composites have been undertaken, more studies are needed to identify novel chitosan blends that can enhance food quality and be used commercially. The stability of chitosan films at elevated temperatures is questionable. However, nanotechnology-based approaches may improve the efficacy of bioactive chitosan films and coatings and aid in developing stable, scalable hybrid materials. Chitosan-based nanocomposites have low toxicity, mucoadhesiveness, regulated release, biodegradability, biocompatibility, and efficient absorption properties, making them a potential biomedical industry candidate.

## Figures and Tables

**Table 1:** The representative applications of various chitosan-based composite in the food and biomedical sectors.

Sr. No.	Chitosan Composite	Application	Ref.
1.	Chitosan and curcumin-based film	The composite improved the shelf life of *Schizothoraxpranati* fillets and prevented protein degradation, delayed lipid oxidation, and the production of volatile amines	[99]
2.	Chitosan nanoparticle-based coating containing cinnamon-perilla essential oils and anthocyanidins	Protected the Red Sea Bream fillets from oxidation and amine development	[100]
3.	Chitosan and tea polyphenols-based film	Delayed the lipid oxidation in shrimps during storage (28 days)	[102]
4.	Chitosan, pomegranate peel extract, and thymus essential oil-based film	Increased the shelf life of fresh beef	[112]
5.	Chitosan, oregano, and thyme essential oils-based film	Inhibited the proliferation of psychrophilic bacteria and increased the shelf life of meat	[114]
6.	Chitosan/nano-titanium dioxide-based film	Decreased deterioration and water loss while delaying respiratory peaks in mangoes	[123]
7.	Chitosan/starch-based film	The film exhibited antibacterial properties and prevented water loss from grapes	[124]
8.	Chitosan/corn starch hydrogel film-embedded extract from *Brassica oleraceae*	Acted as a pH-based sensor and exhibited a distinct color as fish spoils due to its strong sensitivity to pH changes	[131]
9.	Chitosan/PVA polymeric composite	Aided in the regulated release drugs	[144]
10.	Thiolated chitosan and silk fibroin hydrogel	The platform supported chondrocyte proliferation and encouraged the deposition of chondrocyte-specific matrix	[162]
11.	Human cardiac ECM, chitosan, and gelatin composite	Used as a 3D scaffold for a tissue-engineered heart patch	[164]
12.	Chitosan, dextran, and β-glycerophosphate hydrogel loaded with mesenchymal stem cells	Used for the treatment of myocardial infarction	[165]
13.	Chitosan-gallic acid hydrogel	Exhibited wound healing and hemostatic characteristic	[173]

## Data Availability

Not applicable.
